# Sex-related differences in prosthesis-patient mismatch following aortic valve replacement with the edwards intuity valve system

**DOI:** 10.3389/fcvm.2025.1666443

**Published:** 2025-09-04

**Authors:** Muhammad Jawoosh, Rashad Zayat, Leyla Dogan, Yusuf Shieba, Ajay Moza, Lachmandath Tewarie, Shahram Lotfi, Mohammad Amen Khattab, Ahmad Abugameh, Ahmed F. A. Mohammed

**Affiliations:** ^1^ Department of Cardiac Surgery, RWTH University Hospital, Aachen, Germany; ^2^ Faculty of Medicine, RWTH University, Aachen, Germany; ^3^ Department of Cardiothoracic Surgery, Faculty of Medicine, South Valley University, Qena, Egypt; ^4^ Faculty of Medicine, Witten/Herdecke University, Witten, Germany; ^5^ Department of Cardiovascular Surgery, Klinikum Dortmund GGmbH, Dortmund, Germany

**Keywords:** heart valve prosthesis implantation, aortic valve stenosis, heart valve prosthesis, women, echocardiography, prosthesis-patient mismatch, rapid deployment aortic valve replacement

## Abstract

**Background:**

Prosthesis-patient mismatch (PPM) is linked to a poor prognosis following surgical aortic valve replacement (SAVR). The exploration of sex differences in PPM outcomes is currently limited. This study seeks to assess the sex-specific effects of PPM following SAVR was rapid deployment AV (RDAVR) prosthesis the Edwards Intuity.

**Methods:**

From 2018 to 2023, a total of 256 patients (60 females and 196 males) who received isolated or combined RDAVR at our institution were included. The definition of PPM was established through the use of the indexed effective orifice area (EOAi) in accordance with the Valve Academic Research Consortium-3 (VARC-3) criteria. A Multivariate logistic regression was performed to identify predictors of any degree PPM.

**Results:**

Female had higher left ventricular ejection fraction preoperatively (*p* = 0.018). The incidence of any PPM-degree for patients with BMI <30 kg/cm^2^ was significantly higher in female than in male [33 (55%) vs. 26(13.3%), *p* < 0.001]. The same was noted for the incidence of PPM in patients with BMI ≥30 kg/cm^2^ [7 (11.7%) vs. 4 (2.0%), *p* = 0.004]. And the incidence of severe PPM (EOAi ≤0.65 cm^2^/m^2^) for patients with BMI <30 kg/cm^2^ was 16.7% in females vs. 0 in males (*p* < 0.001). The in-hospital mortality did not differ between males and females. In the multivariate logistic regression, we could not identify independent predictors of PPM.

**Conclusions:**

In Patients receiving RDAVR, the incidence of PPM was significantly higher in female than in male. However, we did not find a correlation with early clinical outcomes. The incidence of severe PPM after RDAVR was low in both females and males. Due to differences in geometry and function of the LV in women, further studies are necessary to indicate whether the definition of PPM in men may adhere to elevated EOAi thresholds compared to women.

## 1 Introduction

Aortic valve disease, specifically aortic stenosis (AS), is a significant cardiovascular disease burden, with surgical aortic valve replacement (SAVR) still being the mainstay of treatment even as transcatheter modalities are increasingly being used (1). The Edwards Intuity valve system, a rapid deployment prosthesis including conventional surgical valve implantation with innovative deployment technology, represents a significant advancement in SAVR, potentially decreasing cross-clamp and cardiopulmonary bypass times while maintaining excellent hemodynamic performance (2, 3). However, a critical consideration in valve replacement surgeries is prosthesis-patient mismatch (PPM), which occurs when the effective orifice area (EOA) of the implanted valve prosthesis is too small relative to the patient's body surface area (BSA) and cardiovascular requirements (4, 5).

There is increasing evidence that sex differences are highly significant in the manifestation, course, and treatment outcomes of cardiovascular disease (6). In the context of aortic valve replacement, female patients have distinct anatomical and physiological features which could affect procedural management and results. Females have generally smaller aortic annulus and body surface areas than males and may be at greater risk of PPM following valve replacement (7, 8).

However, the relations between the incidence of PPM and sex with the rapid deployment valves (RDV) for AVR (RDAVR) under investigated. This research aims to study the sex-related differences in clinical outcome and PPM following RDAVR using Edwards Intuity valve system.

## 2 Material and method

### 2.1 Patients' population

A retrospective analysis of prospectively collected data was conducted on all consecutive patients (*n* = 277) who underwent RDAVR with the Intuity prosthesis for symptomatic AS at our hospital from March 2018 to September 2023. The general exclusion criteria for RDAVR at our institution included individuals under 65 years of age or acute endocarditis and for the purpose of this study 21 patients, who already had a cardiac surgery were excluded. RWTH University Ethic committee (EK 151/09) accepted the study procedure, and informed patient consent was obtained.

This study collected patient demographics and assessed perioperative data, including procedural times and rates of procedural and technical success. Discharge evaluations encompassed echocardiography studies, hospital length of stay, ICU length of stay, and in-hospital mortality rates. The rates of early adverse events were analyzed. The adverse events were assessed in accordance with The Society of Thoracic Surgeons Guidelines for reporting mortality and morbidity after cardiac valve interventions (9).

### 2.2 Aortic valve replacement with the intuity prosthesis

According to the surgeon's discretion RDAVR with the EDWARDS INTUITY Valve System was carried out through upper hemi-sternotomy or full sternotomy, as previously delineated by Kocher et al. (3). 

### 2.3 Echocardiography and clinical outcomes

Before surgery and upon hospital discharge, two board-certified physicians conducted thorough transthoracic echocardiograms (TTEs) on patients positioned in the left lateral decubitus posture utilizing commercially available ultrasound machines (GE Vivid E90, GE Vingmed Ultrasound, Horten, Norway) in compliance with established guidelines (10). Pressure gradients, paravalvular leakage, and valve functionality were documented, among other characteristics. Doppler flow velocities were measured from the apical three-chamber perspective utilizing pulsed and continuous wave Doppler modes to evaluate peak and mean gradients. The preoperative aortic valve area (AVA) is calculated by dividing the left ventricular outflow tract (LVOT) stroke volume, determined from the LVOT diameter and velocity time integral, by the aortic valve's velocity time integral (VTI); AVA = (LVOT area × LVOT VTI)/Aortic Valve VTI. Postoperatively, the effective orifice area indexed (EOAi; cm^2^/m^2^) to the body surface area (BSA) of the aortic valve prosthesis was calculated by dividing the EOA by the patient's BSA.

Rapid deployment valve-related complications were characterized as those directly linked to the implantation of the RDV. Valve-related mortality was assessed due to complications arising from RDV implantation, as well as structural or nonstructural valve dysfunction, categorized by paravalvular leakage (PVL) grading (1 = mild; 2 = moderate; 3 = severe) during echocardiography, and the necessity for postoperative pacemaker implantation. Stroke is diagnosed by a neurologist and is defined as a new neurologic deficit confirmed by neuroimaging, such as computed tomography or magnetic resonance imaging.

### 2.4 Prosthesis-patient mismatch definition

We used the VARC-3 criteria to define PPM (11). The absence of PPM is defined independently of sex as EOAi >0.85 cm^2^/m^2^ in patients with a BMI <30 kg/m^2^ or EOAi >0.70 cm^2^/m^2^ in patients with a BMI ≥30 kg/m^2^. Moderate PPM is classified as EOAi between 0.85 and 0.66 cm^2^/m^2^ (BMI <30 kg/m^2^) or EOAi between 0.70 and 0.56 cm^2^/m^2^ (BMI ≥30 kg/m^2^). Severe PPM is defined as EOAi ≤0.65 cm^2^/m^2^ in patients with a BMI <30 kg/m^2^ or EOAi ≤0.55 cm^2^/m^2^ in patients with a BMI ≥30 kg/m^2^.

### 2.5 Statistical analysis

Relevant pre-, peri-, postoperative and echocardiography data were extracted from our institutional database, and statistical analysis was conducted using STATA (StataCorp. 2019. Stata Statistical Software: Release 16) and the jamovi project (2020) (jamovi software, version 2.6.3; https://www.JAMovi.org). Data were presented as mean ± SD or median (range) for continuous variables, as applicable. Categorical variables were presented as absolute counts and proportions (percentages). Continuous variable differences were assessed using unpaired Student's *t*-tests or Mann–Whitney *U*-tests, based on the normality of distribution. Fisher's exact test was employed for categorical variables due to the sample size constraints. Continuous repeating variables were examined using two-way ANOVA to compare both between and within groups. A multivariate binominal logistic regression with the variable any degree of PPM as the dependent variable and alle variables with a *p*-value <0.05 in the univariate analysis [gender, body surface area (BSA), valve size, cardiopulmonary bypass (CPB) time, operation time and the number of performed coronary bypass grafts] as the independent variables, was performed to identify possible factors predicting PPM. A *p*-value below 0.05 was deemed statistically significant.

## 3 Results

### 3.1 Patient demographics and preoperative characteristics

A total of 256 patients who underwent isolated or combined SAVR with the Edwards Intuity valve system were included in this analysis, comprising 196 males (76.6%) and 60 females (23.4%). With exhibition to body BSA (male vs. female: 2.0 ± 0.2 m^2^ vs. 1.8 ± 0.2 m^2^, *p* < 0.001) preoperative demographic data, clinical variables and comorbidities did not significantly differ between males and females Table 1. Despite these differences, the preoperative risk as assessed by European System for Cardiac Operative Risk Evaluation (EuroSCORE) was comparable between males (8% ± 4.1%) and females (8.1% ± 6.5%) (*p* = 0.988) (Table 1).

**Table 1:** Patients’ demographic and preoperative data.

Variable	Male (*n* = 196)	Female (*n* = 60)	Total	*p*
Age years	73.8 (6.0)	74.2 (7.4)	73.9 (6.3)	0.642
BMI kg/m^2^	28.1 (4.5)	27.4 (4.2)	27.9 (4.4)	0.278
BSA m^2^	2.0 (0.2)	1.8 (0.2)	2.0 (0.2)	<0.001
NYHA (I–IV)				
I *n* (%)	3 (1.5)	1 (1.7)	4 (1.6)	0.837
II *n* (%)	13 (6.6)	5 (8.6)	18 (7.1)	
III *n* (%)	176 (89.8)	52 (89.7)	228 (89.8)	
IV *n* (%)	3 (1.5)	0 (0.0)	3 (1.2)	
Diabetes mellitus *n* (%)	71 (36.8)	18 (31.0)	89 (35.5)	0.518
IDDM *n* (%)	8 (4.1)	4 (6.9)	12 (4.8)	0.604
Arterial hypertension *n* (%)	193 (99.5)	58 (100.0)	251 (99.6)	1.000
PHT *n* (%)	23 (11.9)	5 (8.6)	28 (11.1)	0.674
Nicotine abuses *n* (%)	40 (20.6)	6 (10.3)	46 (18.3)	0.113
COPD *n* (%)	16 (8.2)	5 (8.6)	21 (8.3)	1.000
PAD *n* (%)	21 (10.8)	4 (6.9)	25 (9.9)	0.530
Preop dialysis *n* (%)	4 (2.1)	0 (0.0)	4 (1.6)	0.615
Preop AF *n* (%)	41 (20.9)	14 (23.3)	55 (21.5)	0.827
Preop PM *n* (%)	5 (2.6)	0 (0.0)	5 (2.0)	0.474
Prior cardiac surgery *n* (%)	7.8 (5.0)	8.1 (6.5)	7.8 (5.4)	0.679
EuroSCORE mean (SD)	8 (4.1)	8.1 (6.5)	12 (4.8)	0.988
Preoperative echocardiography				
Preop AVA cm^2^	0.8 (0.6, 0.9)	0.6 (0.6, 0.8)	0.8 (0.6, 0.9)	0.004
Preop PPG mmHg	78.0 (67.0, 92.2)	81.5 (66.5, 95.2)	79.5 (67.0, 93.0)	0.224*
Preop MPG mmHg	45.0 (38.0, 54.0)	45.0 (38.0, 54.5)	45.0 (38.0, 54.0)	0.955*
Preop LVEF %	55.0 (40.0, 60.0)	60.0 (50.0, 60.0)	55.0 (45.0, 60.0)	0.018*

Continuous variables presented as mean (SD) or as median (25th, 75th percentile); Categorical variables presented as absolute numbers and percentages. AF, atrial fibrillation; AVA, aortic valve area; BMI, body mass index; NYHA, New York Heart Association; IDDM; insulin dependent diabetes mellitus; COPD, chronic obstructive pulmonary disease; LVEF, left ventricular ejection fraction; PPG, peak pressure gradient; MPG, mean pressure gradient; Preop; preoperative; PHT, pulmonary hypertension; PAD, peripheral arterial disease.

* *p*-value calculated with repeated measurements ANOVA with Tukey adjustment.

Preoperative echocardiographic assessment revealed similar aortic valve gradients between genders (Table 1). While the preoperative left ventricular ejection fraction (LVEF) was significantly lower in males compared to females [55% (40, 60) vs. 60% (50, 60), *p* = 0.018, respectively]. Moreover, the preoperative AVA, was significantly smaller in females than in males [0.6 cm^2^ (0.6, 0.8) vs. 0.8 cm^2^ (0.6, 0.9), *p* = 0.004, respectively] (Table 1).

### 3.2 Operative characteristics and valve size distribution

Intraoperative data revealed significant differences between sexes (Table 2). Operation time was significantly longer for males (169.7 ± 40.7 min) compared to females (156.9 ± 34.6 min) (*p* = 0.031). Similarly, CPB time was significantly longer in males (83.1 ± 24.4 min) than in females (75.4 ± 19.7 min) (*p* = 0.032). Cross-clamp time showed a trend towards longer duration in males (56.0 ± 17.6 min) vs. females (51.3 ± 15.1 min), though this difference did not reach statistical significance (*p* = 0.069). The number of performed coronary artery bypass grafts was significantly higher in males than in females (*p* = 0.031) (Table 2).

**Table 2:** Peri- and postoperative data.

Variable	Male (*n* = 196)	Female (*n* = 60)	Total	*p*
Operation time minutes	169.7 (40.7)	156.9 (34.6)	166.7 (39.7)	0.031
CPB time minutes	83.1 (24.4)	75.4 (19.7)	81.4 (23.6)	0.032
Cross clamp time	56.0 (17.6)	51.3 (15.1)	55.0 (17.2)	0.069
Number of CABG				
1-graft	41 (20.9)	21 (35.0)	62 (24.2)	*0*.*031*
2-grafts	63 (32.1)	21 (35.0)	84 (32.8)	
3-grafts	92 (46.9)	18 (30.0)	110 (43.0)	
Size 19 *n* (%)	0 (0.0)	5 (8.3)	5 (2.0)	<0.001
Size 21 *n* (%)	12 (6.1)	26 (43.3)	38 (14.8)	<0.001
Size 23 *n* (%)	55 (28.1)	23 (38.3)	78 (30.5)	0.176
Size 25 *n* (%)	97 (49.5)	6 (10.0)	103 (40.2)	<0.001
Size 27 *n* (%)	32 (16.3)	0 (0.0)	32 (12.5)	0.002
Any degree PPM&BMI <30	26 (13.3)	33 (55.0)	59 (23.0)	<0.001
Any degree PPM&BMI ≥30	4 (2.0)	7 (11.7)	11 (4.3)	0.004
WI *n* (%)	11 (5.7)	1 (1.7)	12 (4.8)	0.346
Ischemic stroke *n* (%)	9 (4.6)	2 (3.3)	11 (4.3)	0.955
Postop dialysis *n* (%)	3 (1.5)	1 (1.7)	4 (1.6)	1.000
Postop PM *n* (%)	20 (10.2)	3 (5.1)	23 (9.0)	0.345
PRBCs units	0.8 (1.8)	1.5 (2.6)	1.0 (2.0)	0.024
PC units	1.6 (3.0)	2.0 (3.0)	1.7 (3.0)	0.413
FFPs unites	1.0 (2.0)	1.1 (2.2)	1.0 (2.0)	0.649
LOS days	16.7 (8.5)	14.9 (5.7)	16.3 (8.0)	0.128
In-hospital mortality *n* (%)	13 (6.6)	3 (5.0)	16 (6.2)	0.879

Continuous variables presented as mean (SD), Categorical variables presented as absolute numbers and percentages. BMI, body mass index kg/cm^2^; LOS, length of hospital stays; PC, platelets concentrate; PM, pacemaker; PPM, prosthesis-patient mismatch; Postop, postoperative; PRBCs, packed red blood cells; FFPs, fresh frozen plasma, WI, wound infection.

Females required significantly more red blood cells packs than males (1.5 ± 2.6 units vs. 0.8 ± 1.8 units, *p* = 0.024).

The distribution of valve sizes differed significantly between genders (Table 2). Smaller valve sizes were predominantly implanted in females, while larger sizes were more common in males. Size 19 valves were exclusively implanted in females (8.3% vs. 0.0% in males, *p* < 0.001). Size 21 valves were used in 43.3% of females compared to only 6.1% of males (*p* < 0.001). Size 23 valves were used in 38.3% of females and 28.1% of males, though this difference did not reach statistical significance (*p* = 0.176). In contrast, larger valve sizes were predominantly implanted in males: size 25 valves in 49.5% of males vs. 10.0% of females (*p* < 0.001), and size 27 valves exclusively in males (16.7% vs. 0.0% in females, *p* = 0.002) (Table 2).

### 3.3 Postoperative outcomes

The incidence of postoperative complication like the need of pacemaker implantation, ischemic stroke, the need of dialysis and wound infection did not differ between females and males (Table 2).

The hospital length of stay was similar in both males and females (16.7 ± 8.5 days vs. 14.9 ± 5.7, *p* = 0.128, respectively). The rate of in-hospital mortality did not differ between males and females [13 men (6.6%) vs. 3 women (5.0%), *p* = 0.879] (Table 2).

### 3.4 Gender-based variation in valve sizing and postoperative hemodynamics

Postoperatively, LVEF remained lower in males than in females [50% (45, 55) vs. 55% (50, 60), *p* = 0.038, respectively]. The PPG and MPG were significantly higher in females compared to males (*p* = 0.017 and *p* = 0.016, respectively) Table 3. The EOAi measured postoperatively was smaller in female than in male [0.9 (0.8, 1.0) cm^2^ vs. 0.7 (0.6, 0.8) cm^2^, *p* < 0.001, respectively] (Table 3). The incidence of paravalvular leak did not differ between males and females [3 men (1.5%) vs. 0 women, *p* = 0.778].

**Table 3:** Echocardiographic parameter and aortic valve prosthesis performance.

Variable	Male (*n* = 196)	Female (*n* = 60)	*p*
Postop PPG mmHg	15.0 (12.0, 20.0)	16.9 (13.9, 24.4)	*0*.017*
Postop MPG mmHg	8.0 (6.0, 11.0)	9.0 (7.0, 13.0)	*0*.016*
Postop EOAi cm^2^/m^2^	0.9 (0.8, 1.0)	0.7 (0.6, 0.8)	*<0*.*001*
Postop LVEF %	50.0 (45.0, 55.0)	55.0 (50.0, 60.0)	0.038*
Paravalvular leack	3 (1.5)	0 (0.0)	0.778

Continuous variables presented as median (25th, 75th percentile); Categorical variables are presented as absolute numbers and percentages; EOAi, effective orifice area indexed to the body surface area; LVEF, left ventricular ejection fraction; PPG, peak pressure gradient; MPG, mean pressure gradient.

* *p*-value calculated with repeated measurements ANOVA with Tukey adjustment.

Hemodynamic performance also showed differences between males and females. For valve size 21 mm, the PPG and MPG did not differ between females and males but females had a significantly smaller EOAi compared to males (0.70 ± 0.1 cm^2^/m^2^ vs. 0.76 ± 0.1 cm^2^/m^2^, *p* = 0.026). Similarly, for valve size 23 mm, females demonstrated a smaller EOAi (0.8 ± 0.1 cm^2^/m^2^ vs. 0.9 ± 0.1 cm^2^/m^2^, *p* = 0.001), whereas the PPG and MPG did not differ between males and females (Table 4). For the valve size 25 mm we did not detect any differences in the EOAi or the pressure gradients between males and females (Table 4).

**Table 4:** Comparison between female and male categorized according to valve size.

Variable	Male (*n* = 196)	Female (*n* = 60)	*p*-value
Valve size 19	*n* = 0	*n* = 5 (8.33%)	
MPG mmHg		17.8 ± 6.2	
PPG mmHg		31.6 ± 11.6	
EOAi cm^2^/m^2^		0.54 ± 0.05	
BMI kg/m^2^		28.6 ± 5.4	
Valve size 21	*n* = 12 (6.1%)	*n* = 26 (43.33%)	*<0*.*001*
MPG mmHg	10.5 ± 6.9	10.1 ± 4.0	0.836
PPG mmHg	19.5 ± 10.2	18.3 ± 6.4	0.670
EOAi cm^2^/m^2^	0.76 ± 0.1	0.70 ± 0.1	0.026
BMI kg/m^2^	26.4 ± 4.3	26.8 ± 3.9	0.778
Valve size 23	*n* = 55 (28.1%)	*n* = 23 (38.33%)	*0*.*176*
MPG mmHg	8.3 ± 3.6	9.6 ± 3.6	0.140
PPG mmHg	15.8 ± 5.9	18.2 ± 6.5	0.121
EOAi cm^2^/m^2^	0.9 ± 0.1	0.8 ± 0.1	0.001
BMI kg/m^2^	27.5 ± 4.5	27.5 ± 4.6	0.964
Valve size 25	*n* = 97 (49.5%)	*n* = 6 (10%)	*<0*.*001*
MPG mmHg	8.6 ± 3.6	8.2 ± 2.6	0.771
PPG mmHg	15.9 ± 6.0	15.6 ± 4.5	0.899
EOAi cm^2^/m^2^	0.9 ± 0.1	0.9 ± 0.2	0.920
BMI kg/m^2^	28.6 ± 4.7	28.2 ± 2.8	0.836
Valve size 27	*n* = 32 (16.3%)	*n* = 0	
MPG mmHg	8.7 ± 3.1		
PPG mmHg	16.5 ± 6.3		
EOAi cm^2^/m^2^	1.0 ± 0.1		
BMI kg/m^2^	28.2 ± 4.1		

Continuous variables presented as mean (SD). MPG, mean pressure gradient; PPG, peak pressure gradient; EOAi, effective orifice area indexed to body surface area; BMI, body mass index.

### 3.5 Sex-related disparities in PPM based on EOAi thresholds and BMI

The incidence of any degree of PPM for patients with BMI <30 kg/cm^2^ was significantly higher in females than in males [33 (55%) vs. 26 (13.3%), *p* < 0.001]. The same was noted for the incidence of PPM in patients with BMI ≥30 kg/cm^2^ [7 (11.7%) vs. 4 (2.0%), *p* = 0.004].

Further analysis of PPM based on BMI and EOAi thresholds revealed markedly higher rates of PPM among females (Tables 5, 6).

**Table 5:** Prosthesis-patients mismatch according to BMI and EOAi.

Variable	Male (*n* = 196)	Female (*n* = 60)	Total	*p*
Total *n* (%)	196 (76.6)	60 (23.4)	256	
BMI <30 kg/m^2^				
Moderate PPM: 0.66 ≤ EOAi ≤ 0.85	24 (12.2)	23 (38.3)	47 (18.4)	<0.001
Severe PPM: EOAi ≤ 0.65	2 (1.0)	10 (16.7)	12 (4.7)	<0.001
BMI ≥30				
Moderate PPM: 0.56 ≤ EOAi ≤ 70	4 (2.0)	5 (8.3)	9 (3.5)	0.055
Severe PPM: EOAi ≤0.55	0 (0.0)	2 (3.3)	2 (0.8)	0.084

Categorical variables are presented as absolute numbers and percentages. BMI, body mass index; EOAi, effective orifice area indexed to the body surface area; PPM, prosthesis-patient mismatch.

**Table 6:** Distribution of prosthesis-patient mismatch according to BSA and gender.

Variable		no-PPM	Moderate PPM	Severe PPM	Total	*p*
Patients with BMI <30 kg/cm^2^						
Total *n* (%)		130 (68.8)	47 (24.9)	12 (6.3)	189	
BSA m^2^	Mean (SD)	1.9 (0.2)	1.9 (0.3)	1.9 (0.1)	1.9 (0.2)	0.071
Gender	Male *n* (%)	119 (91.5)	24 (51.1)	2 (16.7)	145 (76.7)	<0.001
	Female *n* (%)	11 (8.5)	23 (48.9)	10 (83.3)	44 (23.3)	
Patients with BMI ≥30 kg/cm^2^						
Total *n* (%)		56 (83.6)	9 (13.4)	2 (3.0)	67	
BSA m^2^	Mean (SD)	2.2 (0.2)	2.1 (0.2)	1.8 (0.2)	2.2 (0.2)	0.023
Gender	Male *n* (%)	47 (83.9)	4 (44.4)	0 (0.0)	51 (76.1)	0.001
	Female *n* (%)	9 (16.1)	5 (55.6)	2 (100.0)	16 (23.9)	

BSA, body surface area; PPM, prosthesis-patient mismatch.

**Table 7:** Multivariate logistic regression to predict any degree of PPM.

Model Coefficients – any degree PPM							
						**95% Confidence interval**	
Predictor	Estimate	SE	Z	*p*	Odds ratio	Lower	Upper
Intercept	−1.478	2.465	−0.600	0.549	0.228	0.002	28.587
BSA	−1.287	0.756	−1.703	0.088	0.276	0.063	1.214
Gender							
Female – Male	−0.126	0.410	−0.306	0.759	0.882	0.395	1.970
Valve size	0.151	0.092	1.638	0.101	1.163	0.971	1.393
Op time	−0.009	0.007	−1.341	0.180	0.991	0.979	1.004
CPB time	0.011	0.010	1.003	0.316	1.011	0.990	1.032
n-CABG							
2–3	0.575	0.393	1.462	0.144	1.777	0.822	3.841
1–3	0.753	0.428	1.760	0.078	2.123	0.918	4.908

Note: Estimates represent the log odds of “any degree PPM = PPM” vs. “any degree PPM = no-PPM”.

BSA, body surface area; n-CABG, number of performed coronary bypass grafts; CPB, cardiopulmonary bypass time; OP, operation.

#### 3.5.1 Patients with BMI <30 kg/cm^2^

Overall incidence of moderate PPM (0.66 ≤ EOAi ≤ 0.85 cm^2^/m^2^) was significantly higher in females than in males (38.3% vs. 12.2%, *p* < 0.001, respectively) Table 5. And the incidence of severe PPM (EOAi ≤0.65 cm^2^/m^2^) for patients with BMI <30 was 16.7% in females vs. 1% in males (*p* < 0.001) Table 5.

189 (73.8%) patients had a BMI <30 kg/cm^2^, 145 (76.7%) were male and 44 patients (23.3%) were female. 130 patients (68.8%) did not have PPM. 47 patients (24.9%) had a moderate PPM, of whom 24 patients (51.1%) were male and 23 patients (48.9%) were female. Twelve patients (6.3%) had severe PPM, of whom 10 patients (83.3%) were female (*p* < 0.001) Table 6. The BSA did not differ significantly between patients with severe PPM and patients without PPM in the subgroup patients with BMI <30 kg/cm^2^ (Table 6).

#### 3.5.2 Patients with BMI ≥30 kg/cm^2^

The incidence of moderate or severe PPM in patients with BMI ≥30 kg/cm^2^ did not differ between females and males (Table 5).

67 (26.1%) patients had a BMI ≥30 kg/cm^2^, 51 (76.1%) were male and 16 patients (23.9%) were female. 56 patients (83.6%) did not have PPM. Nine patients (13.4%) had a moderate PPM, of whom 4 patients (44.4%) were male and 10 patients (55.6%) were female. Two patients (3%) had severe PPM, and both of them (100%) were female (*p* < 0.001) Table 6. Patients with BMI ≥30 kg/cm^2^ and severe PPM had significantly lower BSA compared to no-PPM and moderate PPM (1.8 ± 0.2 m^2^ vs. 2.2 ± 0.2 m^2^ vs. 2.1 ± 0.2 m^2^, *p* = 0.023, respectively) Table 6.

### 3.6 Logistic regression with any degree PPM as dependent variable

After entering all variables with a *p*-value <0.05 in the univariate analysis (gender, BSA, valve size, CPB time, operation time and the number of performed coronary bypass grafts) (Table 7) into a multivariate logistic regression with the variable any degree PPM as the dependent variable, none of the variables remained an independent predictor for PPM.

## 4 Discussion

This research examined sex-based variations in baseline characteristics and early postoperative outcomes related to AVR with the Intuity aortic valve prosthesis. The principal findings are as follows: (I) With the exception of a history of prior cardiac surgery, there were no differences in clinical risk profiles between male and female patients at the time of AVR; however, preoperative characteristics of female patients demonstrated a significantly higher LVEF and a smaller native AVA, with no difference in the preoperative pressure gradient; (II) Female patients demonstrated a greater propensity for smaller valve sizes; however, the EOAi was significantly lower in females compared to males, even when using the same valve size in males and females; (III) The prevalence of PPM was greater in females than in males for any EOAi threshold according to the VARC 3-criteria.

In our study, males had prolonged operation time and CPB time which may be attributed to the higher incidence of history of prior cardiac surgeries and the higher number of performed CABG grafts among males.

PPM was initially identified in 1978 by Dr. Rahimtoola (5), who observed that valve prostheses with a reduced EOA appeared to correlate with hemodynamic and clinical deterioration following valve replacement.

The literature presents varying conclusions regarding the relationship between PPM and survival. Numerous single-center studies have indicated no correlation between PPM and reduced survival (12–14), while an equal number have reported the opposite findings (15, 16). Numerous extensive observational meta-analyses have indicated a reduction in survival rates among patients with severe PPM, both overall and within specific subgroups; however, they did not establish a significant correlation for moderate PPM (17, 18). Our Study did not find a correlation between the incidence of PPM and the early postoperative outcome.

As of preoperative risk factors, in our study male patients compared to females are characterized by having a history of prior cardiac surgery and a lower LVEF, similarly to the findings of Steeds et al. (19) They have found that males had a larger incidence of prior cardiac surgery and a reduced LVEF; nevertheless, they also shown an elevated prevalence of renal impairment, increased surgical risk, and a more frequent occurrence of critical preoperative conditions. In contrast to Steeds et al., we did not see significant differences concerning those characteristics.

In our study we found that in patients with BMI ≥30 kg/cm^2^ the BSA was significantly lower in patients who developed severe PPM, these finding are partially in accordance with the large meta-analysis performed by Dayan et al. (20), who included 58 studies with a total number of patients included of 40,381 (39,568 surgical aortic valve replacement and 813 transcatheter aortic valve replacement). Dayan et al. (20), found that the effect of PPM was less significant in patients with a higher body mass index (>28 kg/m^2^) compared to those with a lower index. Factors associated with PPM included advanced age, female gender, hypertension, diabetes, renal failure, increased body surface area, elevated body mass index, and the use of a bioprosthesis. This finding is likely associated with the observation that, in overweight and obese patients, the cardiac output requirement does not rise proportionately with the increase in body surface area resulting from greater body weight. Consequently, the EOA indexed to BSA may exaggerate the extent of PPM in patients with higher BMI (20, 21).

It has been noted that women with comparable hemodynamic AS severity exhibit a reduced level of AV calcification in comparison to men (22). Research indicates that although men experience more favorable outcomes than women following SAVR, various studies demonstrate that women exhibit improved long-term survival rates after transcatheter aortic valve implantation (TAVI) compared to men (23, 24). Various factors have been proposed to explain the sex-related differences in outcomes following aortic valve replacement. One potential factor could be the varying response of the left ventricle to pressure overload in men compared to women (25, 26).

Left ventricular (LV) remodeling in response to pressure overload in AS can be classified into four main patterns (based on LV mass and relative wall thickness) depending on when severe AS is diagnosed: normal geometry, concentric remodeling, concentric hypertrophy, and eccentric hypertrophy (27). In cases of comparable AS severity, women exhibit a more preserved LVEF, reduced left ventricular cavity size, and lower left ventricular mass (index) in comparison to men (26, 28).

Males also were more likely to have larger valve size in comparison to females which can be explained by larger aortic anulus in males.

Interestingly for similar valve sizes (in our study valve size 21 mm, and valve size 23 mm) implanted in men and women the EOAi was lower in females. These findings could be in partly explained as follow: As mentioned above Women exhibited left ventricular concentric remodeling more frequently than men, who more often displayed eccentric left ventricular remodeling (26, 28). That's mean women have more preserved LVEF, reduced left ventricular cavity size, and lower left ventricular mass (index) in comparison to men. Leading to the fact that the measured LVOT diameter in echocardiographic is smaller and as the EOA is calculated as follow: AVA = (LVOT area × LVOT VTI)/Aortic Valve VTI. And the LVOT area is calculated as follow: LVOT Area = π * (LVOT diameter/2)^2^, this will lead to smaller measured EOA and smaller EOAi.

Further analysis of PPM based on BMI and EOAi thresholds revealed markedly higher rates of PPM among females. For patients with BMI <30 kg/cm^2^ and EOAi ≤0.85 cm^2^/m^2^, the incidence of PPM was significantly higher in females (55%) than in males (12.4%) (*p* < 0.001). When the threshold of EOAi ≤0.70 cm^2^/m^2^ for patients with BMI ≥30 kg/cm^2^ was used, PPM was present in 11.7% of females vs. 2.3% of males (*p* = 0.005). These results highlight a considerable sex-based discrepancy in the risk of PPM, with females being much more affected despite similar BMI. This necessitates additional evaluation during the selection and sizing of prostheses in female patients to reduce the likelihood of negative hemodynamic consequences after surgery. However, the incidence of severe PPM remained low for Patients with BMI <30 kg/cm^2^ [males vs. females: 2 (0.9%) vs. 10 (16.7%), *p* < 0.001] and for patients with BMI ≥30 kg/cm^2^ [males vs. females: 0 vs. 2 (3.3%), *p* = 0.066].

These finding are in accordance with Springhetti et al. (29), who analyzed 7,319 patients who underwent SAVR. Springhetti et al. (29) found that any-degree PPM was observed to be more common in women than in men (31.9% vs. 19.7%, *P* < .0001), with a low incidence of severe PPM (2.4% vs. 0.6%, *P* < .0001) as defined by VARC-3 (29). In the study by Springhetti et al. (29), PPM was more common in women than in males and was independently linked to a heightened risk of long-term mortality only in women, as per the VARC-3 classification (11). Springhetti et al. (29) found that after modifying the definition of PPM based on spline-derived EOAi thresholds categorized by sex, PPM demonstrated an independent association with outcomes in all genders.

### 4.1 Limitations

It is important to recognize several limitations. This analysis is retrospective in nature, which introduces certain limitations associated with the study design. Secondly, the study exclusively involved patients with severe AS who were undergoing SAVR with Intuity prosthesis. Consequently, these data may not accurately reflect the characteristics of patients with moderate AS or those undergoing TAVR or SAVR with other prosthesis. Our study suffer the lack of long-term follow-up, and as the PPM correlated with many long-term endpoints, this limit the interpretation of our study.

## 5 Conclusions

In Patients receiving RDAVR, the incidence of PPM was significantly higher in female than in male. However, we did not find a correlation with early clinical outcomes. The incidence of severe PPM after RDAVR was low in both females and males. Due to differences in geometry and function of the LV in women, further studies are necessary to indicate whether the definition of PPM in men may adhere to elevated EOAi thresholds compared to women.

## Data Availability

The original contributions presented in the study are included in the article, further inquiries can be directed to the corresponding author.
